# Exploring the role of gut microbiota in potential mechanism of ketogenic diet in alleviating Parkinson’s disease symptoms

**DOI:** 10.3389/fnins.2026.1678894

**Published:** 2026-01-23

**Authors:** Xian-Mu Luo, Jun Luo, Qian Zhang, Ling-Yong Zeng, Bin Liu, Chun-Ling Chi

**Affiliations:** 1 Department of Cerebrovascular Disease, Mianyang 404 Hospital, Mianyang, China; 2 Department of Neurology, The Fourth Affiliated Hospital of Harbin Medical University, Harbin, China

**Keywords:** clinical trials, gut microbiota, ketogenic diet, mechanism, Parkinson’s disease

## Abstract

**Background:**

Current clinical research suggests that the ketogenic diet (KD) intervention can alleviate Parkinson’s disease (PD) symptoms. However, the underlying mechanisms remain unclear. This pilot study explored the potential link between KD-induced clinical improvements and gut microbiota alterations.

**Methods:**

We engaged 27 PD patients in a 12-week ketogenic dietary trial (16 completed) and employed 16S rRNA sequencing to analyze gut microbiota differences compared to 27 healthy controls.

**Results:**

Baseline analysis revealed distinct dysbiosis in PD patients, characterized by increased abundance of Enterobacteriaceae. Following the 12-week intervention, patients exhibited significant improvements in both motor (MDS-UPDRS Part III, *p* < 0.001) and non-motor symptoms (NMSS, *p* < 0.0001). These clinical improvements were accompanied by specific microbial shifts: a significant increase in *Enterococcus* and Synergistota, and a decrease in *Alloprevotella*.

**Conclusion:**

These findings suggest that the therapeutic effects of the ketogenic diet in PD are associated with specific remodeling of the gut microbiota, particularly the enrichment of potential beneficial taxa and reduction of pro-inflammatory genera.

**Clinical trial registration:**

https://www.chictr.org.cn/index.html, Unique Identifier: ChiCTR2100045394.

## 1 Introduction

Parkinson’s disease (PD) is the second most prevalent neurodegenerative disorder worldwide, affecting approximately 6 million individuals (Steinmetz et al., 2024). The pathological hallmark of PD includes the degeneration of dopaminergic neurons within the nigrostriatal region and the aberrant aggregation of α-synuclein (α-syn) within neural inclusions. However, the underlying etiology of PD remains elusive. To date, no pharmacological interventions have demonstrated efficacy in halting or delaying the progression of PD. Levodopa remains the cornerstone of treatment for managing motor symptoms. However, Levodopa primarily addresses motor symptoms, leaving many non-motor symptoms uncontrolled, and its long-term use is associated with significant side effects such as dose-end and on–off phenomena (Chou et al., 2018). Consequently, many research teams are actively exploring novel PD therapeutic approaches.

The ketogenic diet (KD), a high-fat, low-carbohydrate regimen, has gained attention as a potential neuroprotective intervention. Notably, the ketogenic diet is an established clinical treatment for refractory epilepsy in children (van der Louw et al., 2016). Several clinical researches have recently demonstrated the potential of ketogenic diet intervention as a promising avenue for improving the symptoms of PD (Vanitallie et al., 2005; Tidman et al., 2022). However, the precise mechanism underlying the amelioration of PD symptoms through the ketogenic diet remains unclear. Yet, a study published in *Cell* by Olson et al. introduced a novel perspective (Olson et al., 2018). This study revealed that the ketogenic diet induces alterations in the gut microbiota of seizure mouse models and emphasized the pivotal role of these microbiota changes in conferring protection against seizures. Besides, non-motor symptoms of PD, particularly gastrointestinal dysfunction, often manifest earlier than motor symptoms (Kulcsarova et al., 2023).

Drawing inspiration from the mechanism by which the ketogenic diet modulates gut microbiota to alleviate seizures, we postulate that a similar mechanism may be at play in the context of PD. Notably, no previous investigator has undertaken a comprehensive examination of this hypothesis. Therefore, we conducted a clinical trial to investigate this connection. We recruited PD patients for a 12-week ketogenic diet intervention and analyzed their gut microbiota before and after the diet. Our central hypothesis was that the ketogenic diet alleviates the motor and non-motor symptoms of PD by modulating the gut microbiota, specifically by decreasing the abundance of pro-inflammatory bacteria and increasing the abundance of anti-inflammatory or neuroprotective taxa.

## 2 Patients and methods

### 2.1 Participants

The study was conducted between April 2021 and August 2021, with prior approval from the Ethics Committee of the Fourth Affiliated Hospital of Harbin Medical University (Harbin, China), bearing registration number 2021-WZYSLLSC-04. Additionally, the study was duly registered in the Chinese Clinical Trial Registry (registration number ChiCTR2100045394) before the commencement of participant recruitment. This trial was designed as an exploratory pilot study to assess the feasibility of the intervention and to gather preliminary data on its clinical and microbiological effects. As such, a formal *a priori* power analysis for sample size calculation was not performed, and the initial enrollment target (*n* = 27) was based on feasibility for this intensive dietary protocol.

For PD patients, the inclusion criteria were based on clinical confirmation of primary PD, aligning with the clinical diagnostic criteria of PD published by the Movement Disorders Society in 2015. Exclusion criteria encompassed individuals with a compromised general health status, severe concurrent illnesses, or organ dysfunction. Additionally, individuals who had taken antibiotics within the past 3 months were ineligible for participation.

Healthy control participants (group B) were selected to match the age demographics of the PD patient group and shared the same household environment. Critically, these control participants maintained their regular unrestricted diet throughout the study and did not receive the ketogenic diet intervention. Healthy controls were excluded from the study if they exhibited conditions such as hyposmia, REM sleep behavior disorder, restless legs syndrome, or a Non-Motor Symptoms Questionnaire (NMSQ) score exceeding 3. Individuals taking antibiotics or probiotic supplements within 3 months of sample collection were also excluded from the healthy control group.

### 2.2 Procedure

This trial was designed as a single-arm, pre-post exploratory study, and therefore, patients were not randomized. Following a meticulous screening process, we successfully enrolled a cohort comprising 27 PD patients (group A) and 27 healthy family members (group B). The study employed a single-blind (assessor-blind) design. Due to the nature of the intensive dietary intervention, participants (patients) could not be blinded to the treatment. However, all personnel collecting data and the trained neurologists performing the clinical symptom evaluations remained unaware of the participants’ dietary intervention status throughout the entire study. We subjected both groups to a comprehensive analysis of fundamental demographic parameters, including age, gender, and Hoehn-Yahr Staging (H-Y), with the results presented in Table 1. Stool samples were collected during this pre-intervention phase. To standardize the collection, all participants were instructed to collect the stool samples in the morning (fasted state) to minimize circadian variations. Throughout the 12-week intervention, participants were required to maintain stable dosages of their antiparkinsonian medications to prevent confounding effects on both symptoms and microbiota.

**Table 1:** Baseline characteristics.

Item	Group A (*n* = 27)	Group B (*n* = 27)	*p*
Age (years) Median (p25-p75)	64(59,68)	63 (56.67)	0.116
Male gender n (%)	12(44.4)	10 (37.0)	0.580
H-Y Median (p25-p75)	2(2,2)	——	——

H-Y means Hoehn-Yahr Staging.

In total, 27 PD patients embarked on a 12-week ketogenic diet regimen, with 16 of these patients (designated as group KD) completing the study. Participants followed a strict ketogenic diet (macronutrient ratio: <5% carbohydrates, 75–85% fat, 15–20% protein). To ensure robust compliance, we employed a dual-monitoring strategy. First, a registered dietitian provided continuous supervision through weekly telephone consultations, offering personalized meal guidance and troubleshooting to maintain adherence. Second, objective metabolic compliance was monitored daily using urine ketone test strips. Participants were required to maintain a dipstick result of +++ to +++++, which served as the daily threshold for meeting dietary goals.

Our central hypothesis (as stated in the Introduction) posits that the ketogenic diet alleviates both motor and non-motor symptoms. Therefore, to capture the full spectrum of the diet’s potential effects, we selected a comprehensive battery of validated assessment scales. This multi-domain approach is critical, as many non-motor symptoms (e.g., gastrointestinal, mood, and cognitive issues) are poorly managed by standard dopaminergic therapies and are highly relevant to the gut-brain axis mechanisms investigated in this study. Before and following the ketogenic diet intervention’s implementation, we assessed motor and non-motor symptoms in PD patients while they were in the “on” state.

Motor symptoms were meticulously evaluated employing the Movement Disorder Society-Unified Parkinson’s Disease Rating Scale (MDS-UPDRS) across multiple components,

For the motor domain, symptoms were meticulously evaluated employing the Movement Disorder Society-Unified Parkinson’s Disease Rating Scale (MDS-UPDRS) across multiple components, including Part II, Part III, and Part IV. Additionally, the Schwab and England Activities of Daily Living Scale (S&E) was utilized to gauge the impact of motor symptoms on the patients’ daily functioning.

For the non-motor domain, we administered an array of standardized assessment scales to assess the ketogenic diet’s influence on non-motor symptoms, complications, and disease progression in PD patients. These included MDS-UPDRS Part I, which encompasses non-motor aspects, the Patient Assessment of Constipation Symptoms (PAC-SYM), the Epworth Sleepiness Scale (ESS), the Hamilton Anxiety Scale (HAMA), the Hamilton Depression Scale (HAMD), the Mini-Mental State Examination (MMSE), the Montreal Cognitive Assessment (MoCA), the Non-Motor Symptoms Scale (NMSS), the 39-Item Parkinson’s Disease Questionnaire (PDQ39), the Parkinson’s Disease Sleep Scale (PDSS), the REM Sleep Behavior Disorder Questionnaire (RBDQ-HK), and the Hyposmia Rating Scale (HRS). These comprehensive assessments enabled us to capture a holistic view of both motor and non-motor symptomatology in PD patients undergoing the ketogenic diet intervention.

### 2.3 Stool sample collection, preparation and 16S rRNA sequencing

Bacterial genomic DNA was extracted from stool samples using a commercial kit (Hangzhou Millet: GHFDE100). The V4 hypervariable region of the 16S rRNA gene was amplified using primers 515F/806R and sequenced on an Illumina NovaSeq platform. Samples were sequenced in a single batch to minimize technical variation. The average sequencing depth was 119,690 reads per sample. After quality filtering, raw reads were processed using Vsearch (v2.15.0) and Qiime2 software. To account for uneven sequencing depth, data were rarefied to a depth of 92,590 reads per sample for downstream diversity analyses. Operational taxonomic units (OTUs) were clustered at 97% similarity, and taxonomic annotation was performed against the silva138 database.

### 2.4 Bioinformatic analysis

Analysis of gut microbiota sequence data was performed using QIIME2 and the R package (v3.2.0).

Microbial Diversity Analysis: QIIME2 software was used to calculate α-diversity indices (Chao, Shannon, Simpson) at the OTU level. Differences in α-diversity indices between groups were analyzed using the Wilcoxon rank-sum test, with *p*-values adjusted for multiple comparisons using the Benjamini-Hochberg (FDR) method. Beta-diversity analysis was performed using the UniFrac distance metric and visualized by Principal Component Analysis (PCA) to investigate structural variation across samples.

Group Comparisons of Taxa Abundance: Taxa abundance at all taxonomic levels was statistically compared between groups using the Wilcoxon rank-sum test in R. The resulting *p*-values were corrected for multiple comparisons using the Benjamini-Hochberg false discovery rate (FDR) method.

Biomarker Identification: Linear discriminant analysis effect size (LEfSe), combining LDA with nonparametric tests, was performed to identify differentially abundant taxa across groups using the default parameters (logarithmic LDA score > 2).

Clinical Data Analysis: To compare clinical symptom scores before and after the intervention, the Shapiro–Wilk test was used to assess normality. The paired t-test was used for normally distributed data, and the Wilcoxon matched-pairs signed-rank test was used for non-normally distributed data. Given the multiple scales assessed, all *p*-values from these comparisons were adjusted for multiple testing using the Benjamini-Hochberg false discovery rate (FDR) method.

Significance Threshold: Results were considered statistically significant when the adjusted p-value (q-value) < 0.05.

## 3 Results

### 3.1 Clinical outcomes following the ketogenic diet intervention

**Figure 1:**
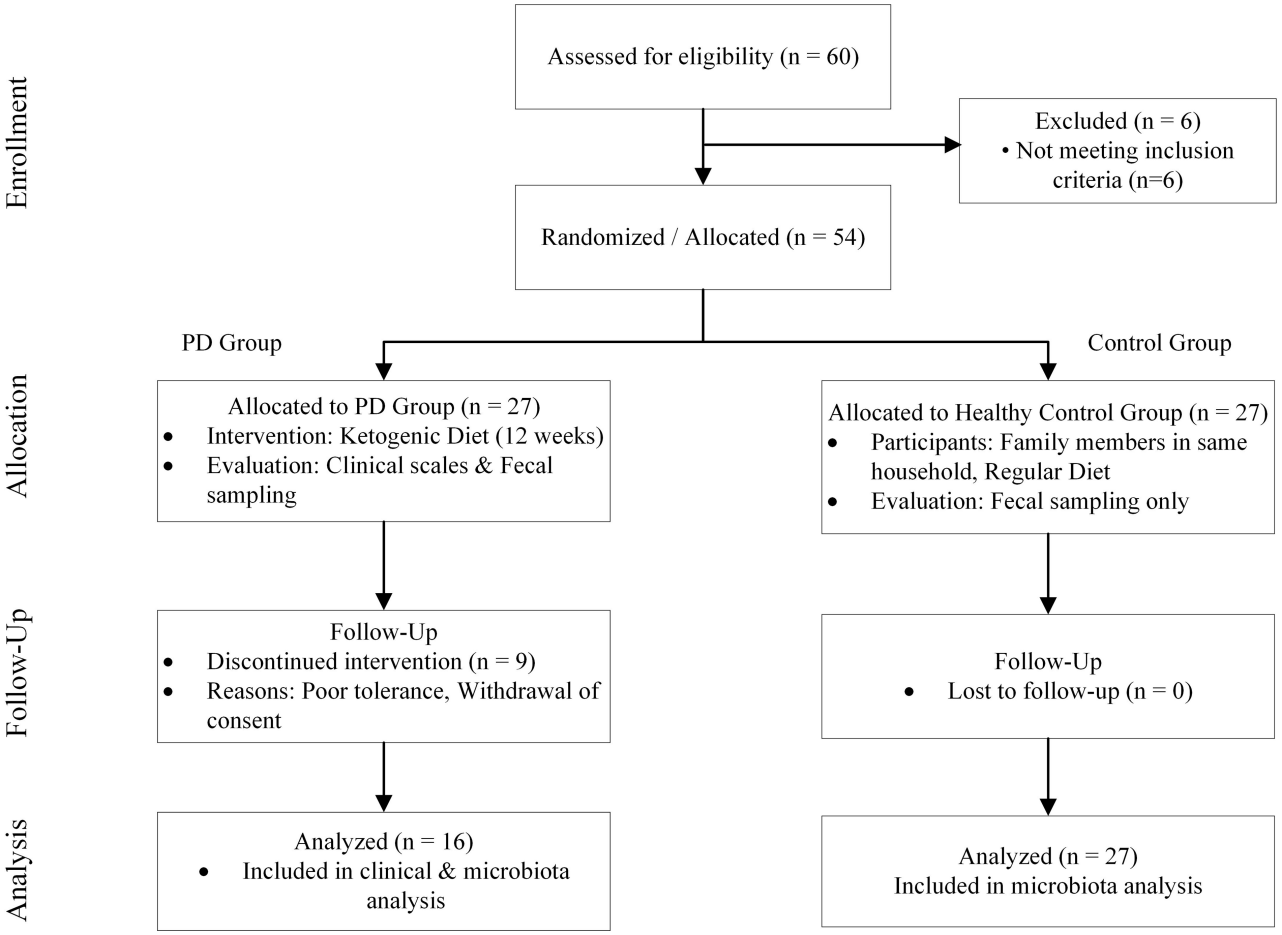
Consort flow diagram of the study design. The diagram illustrates the recruitment, allocation, and follow-up of participants. Patients with Parkinson’s disease (PD) underwent a 12-week ketogenic diet intervention, while family-matched healthy controls maintained their regular unrestricted diet to serve as a baseline for gut microbiota comparison.

Of the 27 patients with Parkinson’s disease initially enrolled in the intervention, 16 (59.3%) successfully completed the 12-week ketogenic diet (Figure 1). The remaining 11 participants withdrew from the study prior to its completion. The reasons for withdrawal included difficulty adapting to the new dietary regimen (*n* = 9), one participant who was unable to tolerate the associated hunger (*n* = 1), and another who cited a lack of family cooperation for the dietary changes (*n* = 1). It is noteworthy that no other significant adverse side effects were reported during the process.

To evaluate the clinical efficacy of the ketogenic diet, we assessed changes in motor and non-motor symptoms in the 16 PD patients who completed the 12-week intervention. Pre- and post-intervention scores from a comprehensive set of standardized scales were compared, with the results summarized in Table 2.

**Table 2:** Changes in clinical symptoms in PD patients after 12-week ketogenic diet intervention.

Assessment Scale	*p*-value
MDS-UPDRS Part III (Total Motor Score)	< 0.001
MDS-UPDRS Part II (Motor Experiences of Daily Living)	< 0.0001
S&E (Activities of Daily Living)	0.002
MDS-UPDRS Part I (Non-Motor Experiences of Daily Living)	< 0.001
NMSS (Non-Motor Symptoms Scale)	< 0.0001
PAC-SYM (Constipation)	0.002
ESS (Daytime Sleepiness)	0.002
HAMA (Anxiety)	< 0.001
HAMD (Depression)	< 0.0001
RBDQ-HK (REM Sleep Behavior Disorder)	0.002
PDQ-39 (Quality of Life)	0.002
MMSE (Cognition)	0.004
MoCA-B (Cognition)	< 0.001
PDSS (Sleep Scale)	0.076
HRS (Hyposmia)	0.182

Data represent comparisons of scores before and after the 12-week intervention in 16 PD patients. Statistical significance was assessed using paired t-test or Wilcoxon matched-pairs signed-rank test as appropriate. *p* < 0.05 was considered statistically significant. For all scales except MMSE and MoCA-B, a score decrease indicates improvement. For MMSE and MoCA-B, a score increase indicates improvement. MDS-UPDRS, Movement Disorder Society-Unified Parkinson’s Disease Rating Scale; S&E, Schwab and England Activities of Daily Living Scale; NMSS, Non-Motor Symptoms Scale; PAC-SYM, Patient Assessment of Constipation Symptoms; ESS, Epworth Sleepiness Scale; HAMA, Hamilton Anxiety Scale; HAMD, Hamilton Depression Scale; RBDQ-HK, REM Sleep Behavior Disorder Questionnaire-Hong Kong; PDQ-39, 39-Item Parkinson’s Disease Questionnaire; MMSE, Mini-Mental State Examination; MoCA-B, Montreal Cognitive Assessment-Basic; PDSS, Parkinson’s Disease Sleep Scale; HRS, Hyposmia Rating Scale.

Overall, patients demonstrated substantial improvements in motor function. The total motor score, as measured by the MDS-UPDRS Part III, decreased significantly (*p* < 0.001). Furthermore, there was a highly significant improvement in motor experiences of daily living, as indicated by the decrease in MDS-UPDRS Part II scores (*p* < 0.0001) and the increase in the Schwab and England Activities of Daily Living Scale scores (*p* = 0.002).

The ketogenic diet also led to broad enhancements in non-motor symptoms. Significant improvements were recorded for overall non-motor experiences of daily living (MDS-UPDRS Part I, *p* < 0.001) and the total Non-Motor Symptoms Scale (NMSS, *p* < 0.0001). Specifically, patients experienced significant relief from constipation (PAC-SYM, *p* = 0.002), excessive daytime sleepiness (ESS, *p* = 0.002), anxiety (HAMA, *p* < 0.001), depression (HAMD, *p* < 0.0001), and REM sleep behavior disorder (RBDQ-HK, *p* = 0.002). Correspondingly, overall quality of life improved significantly (PDQ-39, *p* = 0.002). Notably, cognitive function was enhanced, evidenced by significant increases in both MMSE (*p* = 0.004) and MoCA-B scores (*p* < 0.001). In contrast, the intervention did not significantly impact overall sleep quality as measured by the PDSS (*p* = 0.076) or hyposmia as measured by the HRS (*p* = 0.182).

### 3.2 Differences in gut microbiota between PD patients and healthy controls

At baseline, analysis of 16S rRNA gene sequencing revealed significant differences in the gut microbiota between PD patients (group A) and healthy controls (group B). Specifically, the overall community structure (*β*-diversity) was distinct between the two groups. Additionally, the α-diversity was higher in PD patients compared to the healthy group (Simpson index, *p* = 0.027).

**Figure 2:**
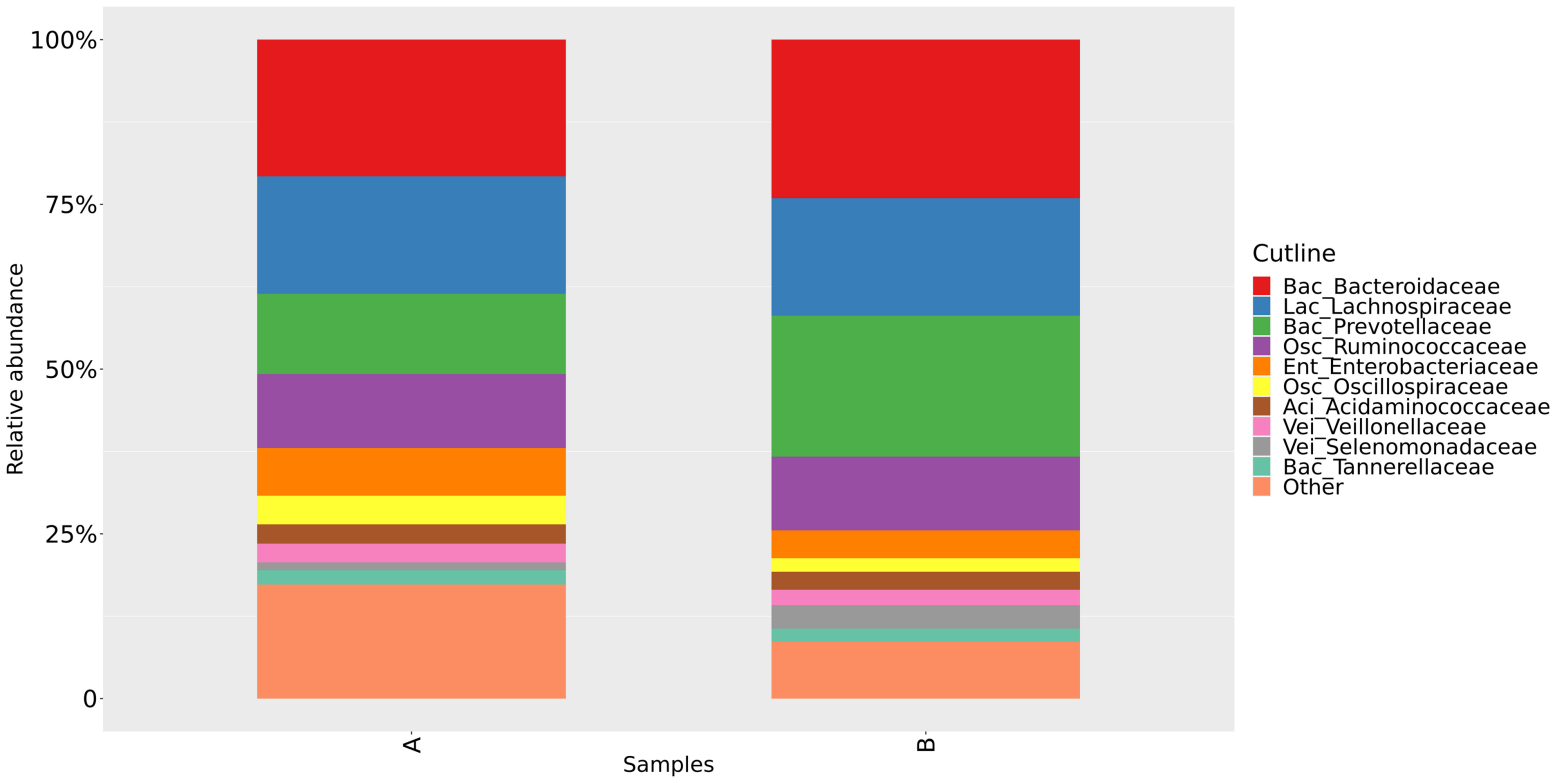
Relative abundance of gut microbiota in group A and group B at the family level. Group A: PD patients before the ketogenic diet. Group B: healthy controls.

**Figure 3:**
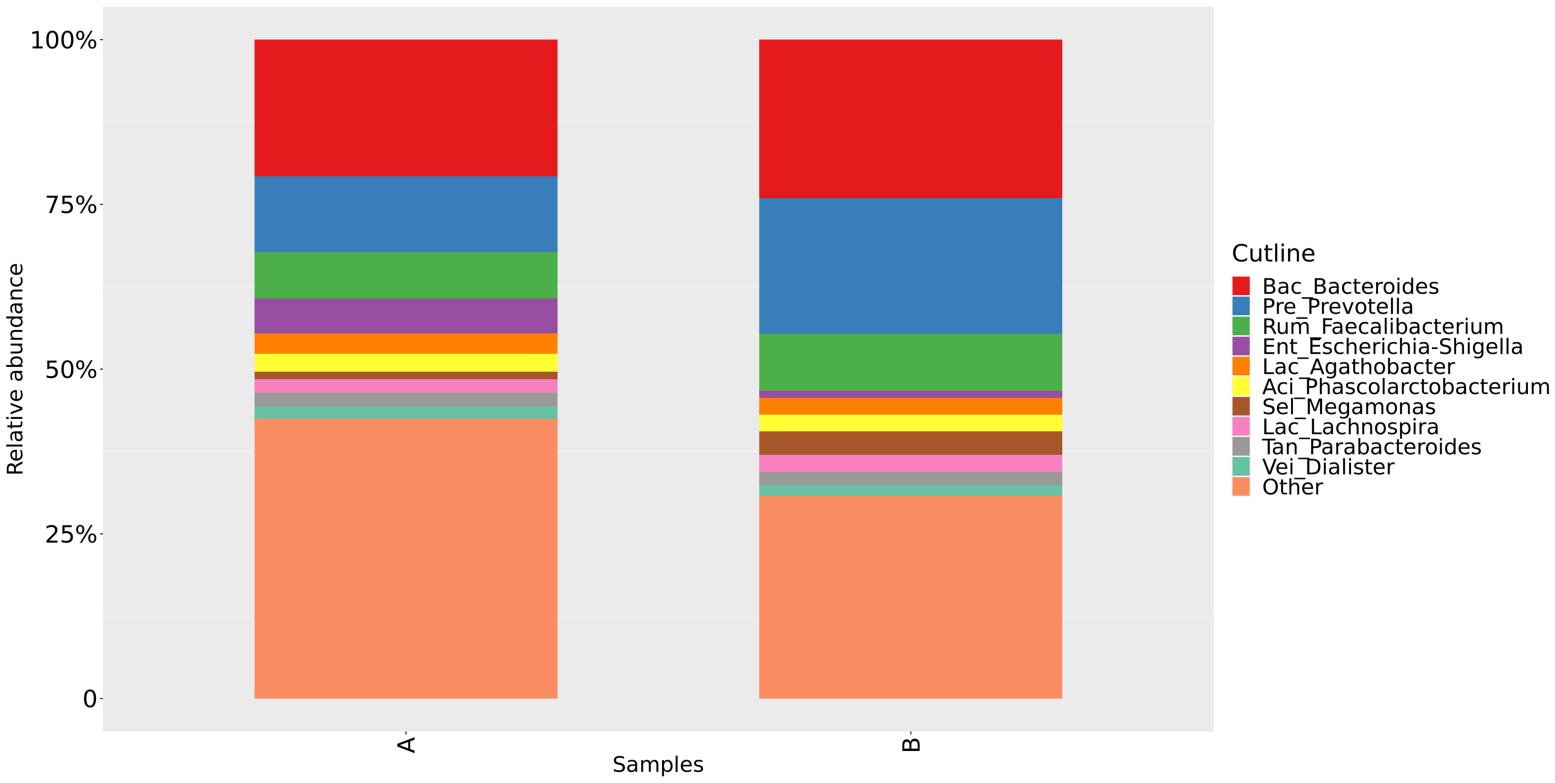
Relative abundance of gut microbiota in group A and group B at the genus level. Group A: PD patients before the ketogenic diet. Group B: healthy controls.

We could confirm a difference in gut microbiota composition between group A and group B, as shown in Figures 2 and 3. At the family level, our study showed that the relative abundance of Bacteroidaceae and Prevotellaceae was reduced. However, Enterobacteriaceae and Oscillospiraceae were increased in group A. Moreover, at the genus level, we found that the relative abundance of *Bacteroides*, *Prevotella* and *Faecalibacterium* decreased, while *Escherichia-Shigella* increased significantly in group A compared to group B.

**Figure 4:**
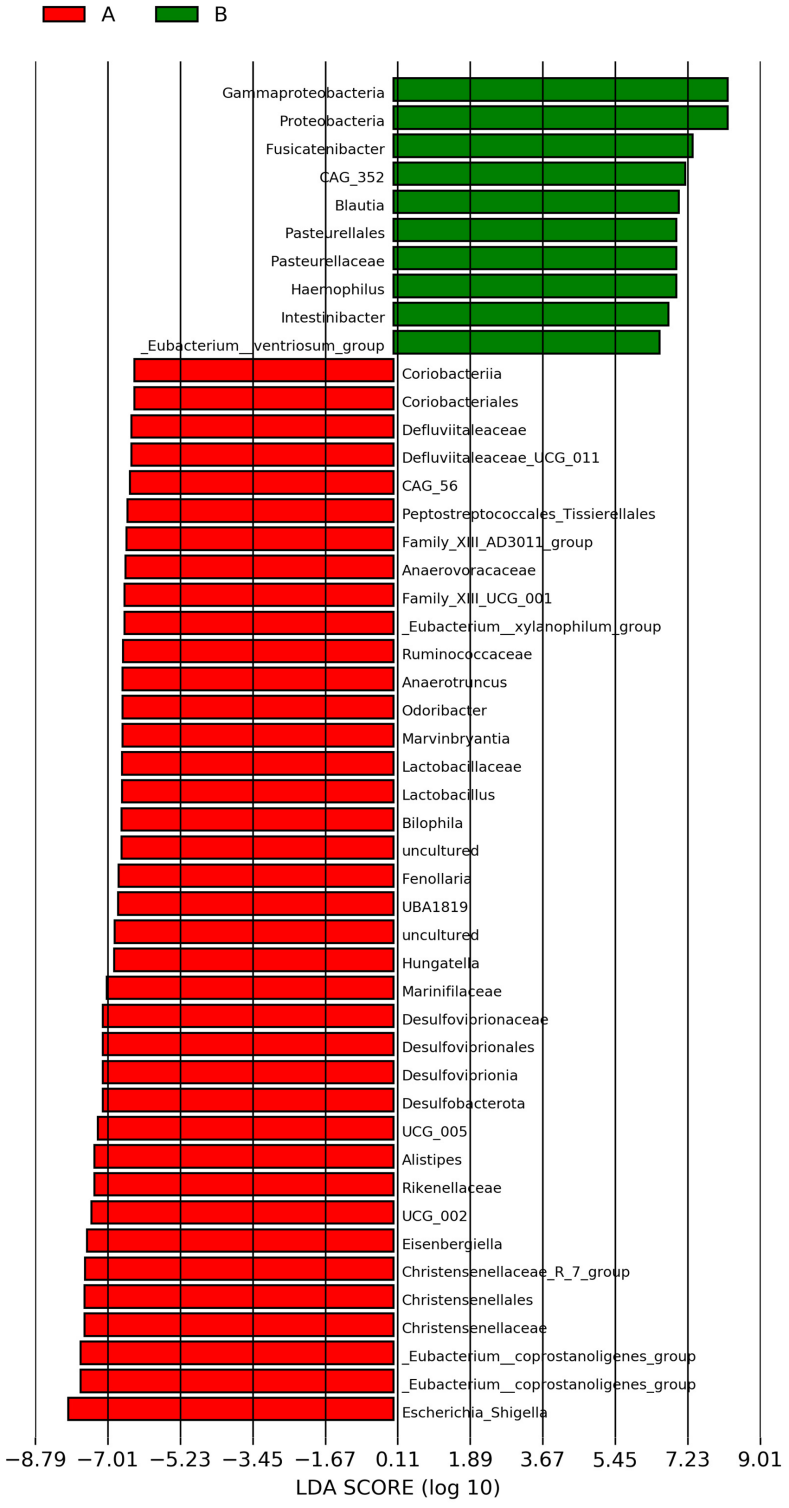
LEfSe analysis of group A and group B. Thresholds used for inclusion: linear discriminant analysis (LDA) score > 2.0 and FDR-adjusted *p* < 0.05. Group A: PD patients before the ketogenic diet. Group B: healthy controls. As detailed in the iscussion, roup A (red) shows enrichment in taxa often associated with inflammation (e.g., Enterobacteriaceae, Lactobacillaceae), while roup B (green) is enriched in taxa associated with SCFA production and gut health (e.g., *Bacteroides*, *Roseburia*).

LEfSe analysis revealed significant microbial species differences between groups A and B (LDA score > 2.0, *p* < 0.05). The results of LEfSe analysis showed that a total of 38 unique microbial species differed between group A and group B, of which 31 were in group A and 7 in group B. Among those, Lactobacillaceae, *Lactobacillus*, *Dorea*, *Alistipes*, *Odoribacter* and Enterobacteriaceae were the dominant groups in group A, while Bacteroidota, *Bacteroides*, *Roseburia* and Pasteurellaceae are the dominant groups in group B, as shown in Figure 4.

### 3.3 Changes in gut microbiota before and after ketogenic diet in PD patients

Following the 12-week ketogenic diet intervention, however, we observed no significant changes in either the α- or β-diversity of the gut microbiota in PD patients (group KD) compared to their pre-intervention state (group A). This suggests that the observed clinical benefits are likely driven by targeted shifts in the abundance of specific key bacteria rather than a large-scale restructuring of the entire microbial community.

**Figure 5:**
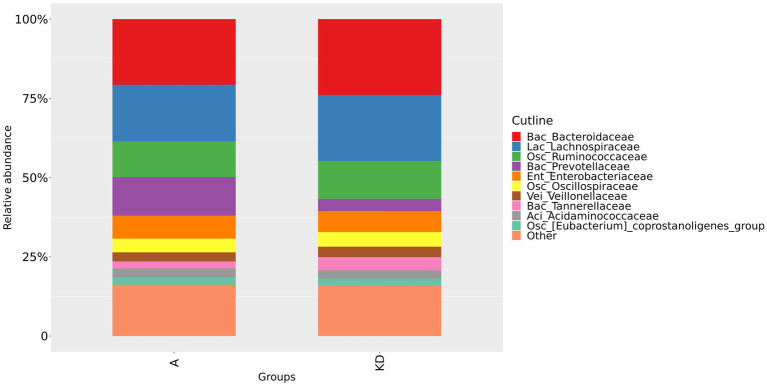
Relative abundance of gut microbiota in group A and group KD at the family level. Group A: PD patients before the ketogenic diet. Group KD: PD patients after the ketogenic diet.

**Figure 6:**
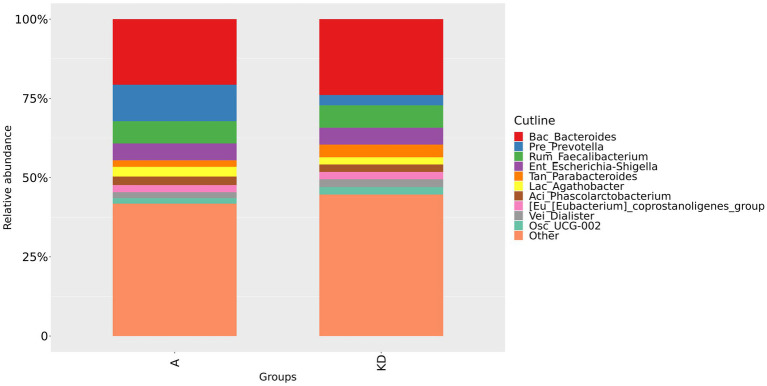
Relative abundance of gut microbiota in group A and group KD at the genus level. Group A: PD patients before the ketogenic diet. Group KD: PD patients after the ketogenic diet.

The relative abundance of gut microbiota in PD patients was altered after the ketogenic diet. At the family level, the relative abundance of Bacteroidaceae, Lachnospiraceae, and Tannerellaceae increased in the group KD; however, the relative abundance of Prevotellaceae decreased, as shown in Figure 5. At the genus level, the relative abundance of *Bacteroides*, *Parabacteroides* and *Dialister* in group KD tended to increase, but *Prevotella* and *Agathobacter* reduced after the ketogenic diet, as shown in Figure 6. These results indicated that overall microbial composition at the family and genus levels differed significantly between the two groups.

### 3.4 Transformation in dominant gut microbiota following ketogenic diet in PD patients using LefSe

**Figure 7:**
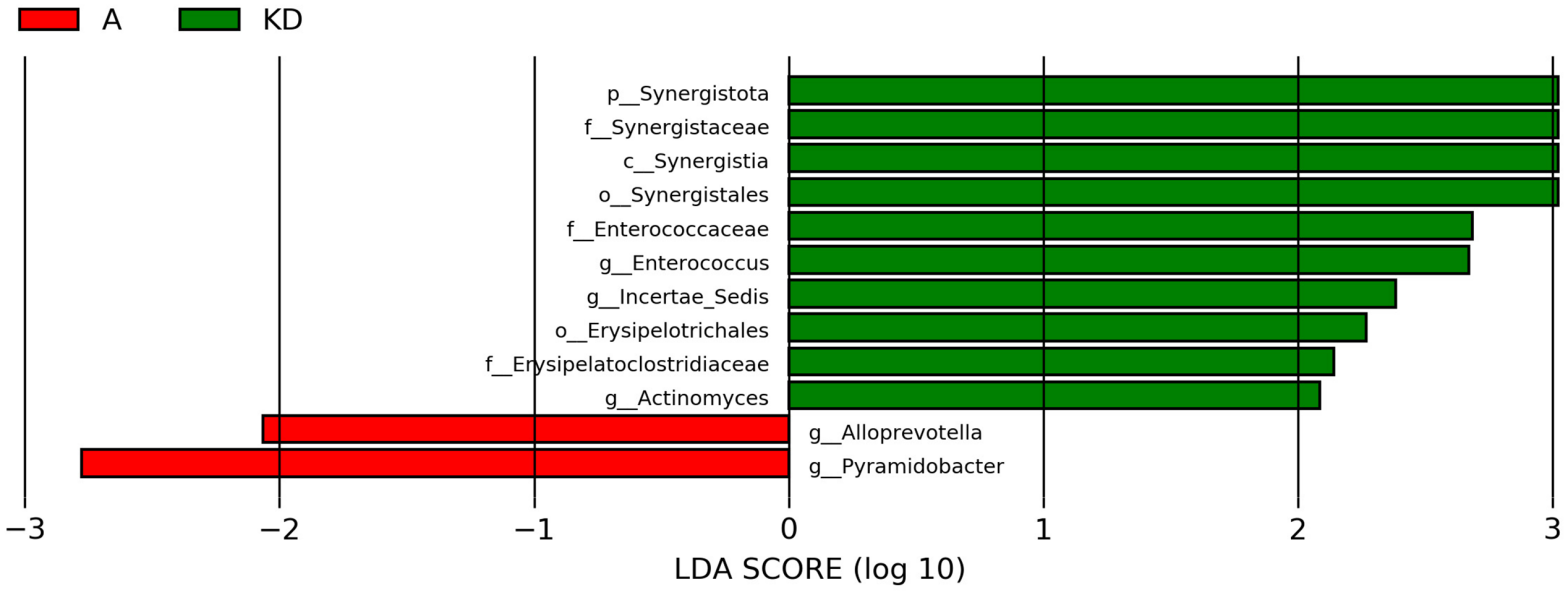
LEfSe analysis of group A and group KD. Thresholds used for inclusion: Linear Discriminant Analysis (LDA) score > 2.0 and FDR-adjusted *p* < 0.05. Group A: PD patients before the ketogenic diet. Group KD: PD patients after the ketogenic diet. This plot illustrates the diet-induced shift. Group A (pre-KD, red) was enriched in taxa such as *Alloprevotella* (linked to inflammation), while group KD (post-KD, green) showed significant enrichment in taxa like *Enterococcus* and *Synergistota*, which are discussed for their potential anti-inflammatory and beneficial roles.

To further determine whether specific individual bacterial taxa were differentially enriched between group A and group KD, we applied LEfSe analysis. As shown in Figure 7, The following 12 taxa showed differentiated distributions with LDA scores >2: Synergistota, Synergistaceae, Synergistia, Synergistales, Enterococcaceae, *Enterococcus*, incertae_sedis, Erysipelotrichales and *Actinomyces* were significantly more abundant in fecal samples from the group KD, while we identified significant enrichment in *Alloprevotella* and *Pyramidobacter* in group A.

## 4 Discussion

The most important finding of our study was that a 12-week ketogenic diet intervention improved motor and non-motor symptoms in PD patients. Additionally, the ketogenic diet affected the gut microbiota of PD patients.

### 4.1 Pathways of gut microbiota involving in PD

Our study confirmed that the gut microbiota composition in PD patients is altered compared to healthy controls, a finding consistent with a growing body of literature. These baseline differences provided the context for investigating the effects of the ketogenic diet (Barichella et al., 2019; Qian et al., 2020; Zhang et al., 2020).

A key feature of this dysbiosis is the depletion of beneficial bacteria crucial for gut health. For instance, we observed a significant reduction in Bacteroidota and *Roseburia* in PD patients, which aligns with previous studies (Li et al., 2017; Vidal-Martinez et al., 2020). The phylum Bacteroidota includes major producers of propionate (Reichardt et al., 2014), a short-chain fatty acid (SCFA) that has shown neuroprotective effects by rescuing motor deficits in PD mouse models (Duan et al., 2024). Similarly, *Roseburia* is a key butyrate producer, and butyrate is known to protect the intestinal barrier, exert anti-inflammatory effects, and prevent dopaminergic neuronal degeneration (Krueger et al., 2025). The loss of these SCFA-producing bacteria likely contributes to the impaired gut barrier integrity and chronic inflammation associated with PD (Parada et al., 2019). However, it is important to note that we did not quantify fecal SCFA levels in this study, so this functional consequence is inferred from the taxonomic data.

Concurrently with the loss of beneficial microbes, our PD cohort showed an enrichment of potentially pro-inflammatory bacteria. Notably, the relative abundance of Enterobacteriaceae and Lactobacillaceae was significantly higher in PD patients (Hopfner et al., 2017). Increased Enterobacteriaceae has been positively correlated with the severity of postural instability and gait difficulty in PD (Scheperjans et al., 2015) and is a known source of endotoxin (LPS), which can promote neuroinflammation (Menozzi et al., 2025). Although some *Lactobacillus* species are considered probiotics, increased abundance of the Lactobacillaceae family in PD has also been linked to heightened inflammatory markers and may be influenced by levodopa use (Maini et al., 2019; van Kessel et al., 2019).

Taken together, this dysbiotic signature—characterized by a deficit in neuroprotective SCFA producers and an overgrowth of pro-inflammatory pathobionts—presents a plausible mechanism through which the gut microbiota is involved in the pathogenesis of PD. This state of chronic, low-grade intestinal inflammation can compromise the integrity of both the intestinal wall and the blood–brain barrier, creating a permissive environment for neurodegenerative processes (Li et al., 2021). Understanding this altered landscape is crucial, as it sets the stage for therapeutic interventions like the ketogenic diet, which aims to modulate this very ecosystem to achieve clinical benefits.

### 4.2 Potential mechanisms of ketogenic diet improving PD through gut microbiota

Although several studies have reported that ketogenic diet intervention improves PD symptoms, there is no experimental evidence indicating whether this improvement is associated with changes in gut microbiota. Therefore, our trial also focused on the effect of the ketogenic diet on the gut microbiota of PD patients.

Interestingly, the 12-week ketogenic diet intervention did not lead to significant changes in overall gut microbial diversity in our PD patients, a finding also reported in studies of ketogenic diet for epilepsy (Lindefeldt et al., 2019). This supports our hypothesis that the clinical improvements are not driven by a large-scale restructuring of the entire gut microbial ecosystem but rather by the selective modulation of specific key taxa. Our finding also suggests that the clinical improvements observed in our cohort are not necessarily driven by a large-scale restructuring of the entire gut microbial ecosystem. Rather, it implies a more nuanced mechanism, whereby the ketogenic diet selectively modulates the abundance of specific key taxa that play a critical role in host–microbe interactions. This perspective, therefore, elevates the importance of the specific taxonomic shifts we identified—such as the increase in potentially beneficial bacteria and the reduction in pro-inflammatory genera—as potential contributors to the therapeutic effect.

Moreover, the ketogenic diet impacts the composition of the gut microbiota of PD patients, as we will discuss below. After 12 weeks of ketogenic diet intervention, we observed a significant increase in *Enterococcus* and Synergistota while a decrease in *Alloprevotella* in PD patients. *Enterococcus faecalis* and *Enterococcus faecium* are the most predominant species of *Enterococcus* genus. Previous study found that transplantation of *Enterococcus faecalis* and *Enterococcus faecium* into the intestine of PD mice increased striatal dopamine levels in the mouse brain and improved symptoms of PD (Wang et al., 2021). In cellular experiments, LPS-induced elevated TNF-*α* levels were significantly suppressed upon exposure to *Enterococcus faecalis*, demonstrating a significant anti-inflammatory effect of *Enterococcus faecalis*; in a mouse model, young mice supplemented orally with *Enterococcus faecalis* for 28 days exhibited reduced oxidative markers and enhanced antioxidant enzyme activity in the brain, along with increased levels of *γ*-aminobutyric acid and dopamine (Divyashri et al., 2015). Therefore, based on these preclinical findings, we speculate that the improvement in clinical symptoms in our PD patients might be potentially linked to the observed elevation of *Enterococcus* and its dopamine-regulatory capacity. However, since we did not directly measure dopamine levels in this cohort, this mechanistic link remains hypothetical and requires validation in future human studies. Regarding the post-intervention shifts, the observed increase in Synergistota may be primarily a response to the dietary substrate. Previous studies have linked Synergistota abundance to increased cholesterol intake, which is inherent to the high-fat ketogenic diet (Nuli et al., 2019). While its specific role in PD remains to be fully elucidated, its enrichment confirms a distinct modulation of the microbiome by the dietary regimen.

In contrast, the relative abundance of *Alloprevotella* significantly decreased. Elevated *Alloprevotella* has been previously associated with gut inflammation and cognitive impairment (Gao et al., 2021; Xu et al., 2021; Du et al., 2022; Kaiyrlykyzy et al., 2022). Given that our participants showed significant improvements in cognitive function (MoCA scores) and non-motor symptoms, the reduction of this potentially pro-inflammatory genus may reflect a beneficial shift in the gut-brain axis environment.

To sum up, the ketogenic diet intervention altered the gut microbiota of PD patients. This microbial shift coincided with clinical improvements, suggesting a potential role for gut-brain axis pathways involving anti-inflammation and dopamine regulation.

Beyond these mechanistic insights, our findings carry significant clinical implications. The 12-week ketogenic diet demonstrated a robust, broad-spectrum therapeutic effect. Crucially, it improved not only motor function but also a wide array of non-motor symptoms (NMS)—including anxiety, depression, constipation, and cognitive function. These NMS are often refractory to standard dopaminergic therapies and are a major determinant of patients’ quality of life. Our results position the KD as a powerful, non-pharmacological adjunctive therapy for holistic PD management. Furthermore, by linking these clinical gains to specific microbial shifts (such as the reduction of *Alloprevotella*), this study provides a tangible rationale for future interventions that may move beyond the diet itself to directly target these microbial pathways.

### 4.3 Limitations of the study

We acknowledge several limitations in this study. First and foremost, the final sample size (*n* = 16) is small. As an exploratory pilot study, no *a priori* power calculation was performed. Although we initially enrolled 27 patients, the 12-week ketogenic diet proved challenging, leading to a high dropout rate (40.7%) primarily due to difficulties in adapting to the regimen. We acknowledge that this high attrition introduces potential selection bias, as the final cohort likely represents a subgroup of patients with higher motivation or better caregiver support. Besides, due to the preliminary nature of data collection in this pilot phase, comprehensive baseline data for dropouts was not available for statistical comparison with completers. Therefore, we could not perform an intent-to-treat (ITT) analysis, and attrition bias cannot be definitively excluded based on quantitative metrics. This limits the generalizability of our findings to the broader PD population. Additionally, we caution that due to the small sample size, precise point estimates of the magnitude of clinical improvement (effect sizes) may be subject to variability. Therefore, we focused on reporting statistical significance to demonstrate proof-of-concept, while future larger trials are needed to accurately quantify the clinical effect sizes.

Furthermore, this study lacked a parallel control group of PD patients who did not receive the ketogenic diet intervention. Our healthy control group (group B) was designed to establish baseline differences in microbiota compared to PD patients, rather than to assess the intervention’s efficacy. We therefore acknowledge that without a non-intervention PD control group, we cannot definitively separate the specific effects of the diet from potential placebo effects or spontaneous symptom variations.

Third, the 12-week duration of our trial only allowed for the assessment of short-term effects. We were unable to evaluate the long-term sustainability of either the clinical improvements or the dietary adherence itself. Although the study was conducted in 2021, systematic long-term follow-up was not part of the original study design. This remains a critical question for future research.

Furthermore, while participants were required to maintain stable antiparkinsonian medication regimens during the trial, we acknowledge the potential confounding effects of these drugs (e.g., Levodopa) on gut microbiota composition. In a single-arm study without a non-diet control group taking similar medications, the specific impact of the diet versus the medication cannot be fully disentangled.

Finally, this study provides correlational evidence rather than direct mechanistic proof. We acknowledge that the biological interpretation of specific taxonomic shifts observed in this small pilot cohort is limited and requires validation in larger, independent cohorts. As the reviewer noted, we did not measure key potential mediators such as short-chain fatty acids (SCFAs) or systemic inflammatory markers. Our discussion on these mechanismsis therefore based on the known functions of the bacteria we identified, rather than on direct measurement. Future studies must incorporate these analyses to confirm the mechanisms hypothesized here.

Nevertheless, we strongly agree that these promising results must be validated in future, large-scale, multi-center, and long-term clinical trials (ideally including a randomized, non-intervention PD patient control arm) that also integrate these crucial mechanistic measurements.

## 5 Conclusion

This study marks the first attempt to link the mechanisms underlying the improvement of alleviation of PD by the ketogenic diet and gut microbiota. Our experiments have revealed that PD patients exhibit alterations in the diversity and composition of their gut microbiota compared to the healthy control group. Within the gut, there is an increase in the abundance of bacteria known to promote inflammation, reduce SCFAs, and contribute to neuronal degeneration. These changes may represent potential mechanisms through which gut microbiota may be involved in the onset of PD. Ketogenic diet intervention induces shifts in the gut microbiota of PD patients, characterized by an increase in the abundance of anti-inflammatory bacteria and a decrease in bacteria associated with pro-inflammatory responses. Consequently, it is plausible to speculate that the ketogenic diet exerts its effects by influencing the gut microbiota. This study provides valuable insights that could serve as a theoretical foundation for the clinical implementation of the ketogenic diet as a potential novel therapy. Future investigations should employ multi-omics approaches, including shotgun metagenomics and metabolomics, to validate the functional implications of these microbial shifts. Specifically, directly quantifying SCFAs, inflammatory markers, and other key metabolites in larger, randomized controlled trials will be essential to confirm the deeper mechanistic links proposed in this pilot study.

## Data Availability

The data presented in the study are deposited in the Scientific Data Bank (ScienceDB), accession links: https://www.scidb.cn/s/zqyuua.

## References

[ref1] Barichella M. Severgnini M. Cilia R. Cassani E. Bolliri C. Caronni S. . (2019). Unraveling gut microbiota in Parkinson's disease and atypical parkinsonism. Mov. Disord. 34, 396–405. doi: 10.1002/mds.27581, 30576008

[ref2] Chou K. L. Stacy M. Simuni T. Miyasaki J. Oertel W. H. Sethi K. . (2018). The spectrum of “off” in Parkinson's disease: what have we learned over 40 years? Parkinsonism Relat. Disord. 51, 9–16. doi: 10.1016/j.parkreldis.2018.02.001, 29456046

[ref3] Divyashri G. Krishna G. Muralidhara Prapulla S. G. (2015). Probiotic attributes, antioxidant, anti-inflammatory and neuromodulatory effects of *Enterococcus faecium* CFR 3003: in vitro and in vivo evidence. J. Med. Microbiol. 64, 1527–1540. doi: 10.1099/jmm.0.000184 26450608

[ref4] Du Y. Li X. An Y. Song Y. Lu Y. (2022). Association of gut microbiota with sort-chain fatty acids and inflammatory cytokines in diabetic patients with cognitive impairment: a cross-sectional, non-controlled study. Front. Nutr. 9:930626. doi: 10.3389/fnut.2022.930626, 35938126 PMC9355148

[ref5] Duan W. Wang F. Liu J. Liu C. (2024). Relationship between short-chain fatty acids and Parkinson’s disease: a review from pathology to clinic. Neurosci. Bull. 40, 500–516. doi: 10.1007/s12264-023-01123-9, 37755674 PMC11003953

[ref6] Gao R. Tian S. Wang J. Zhu W. (2021). Galacto-oligosaccharides improve barrier function and relieve colonic inflammation via modulating mucosa-associated microbiota composition in lipopolysaccharides-challenged piglets. J. Anim. Sci. Biotechnol. 12:92. doi: 10.1186/s40104-021-00612-z, 34376253 PMC8356462

[ref7] Hopfner F. Künstner A. Müller S. H. Künzel S. Zeuner K. E. Margraf N. G. . (2017). Gut microbiota in Parkinson disease in a northern German cohort. Brain Res. 1667, 41–45. doi: 10.1016/j.brainres.2017.04.019, 28506555

[ref8] Kaiyrlykyzy A. Kozhakhmetov S. Babenko D. Zholdasbekova G. Alzhanova D. Olzhayev F. . (2022). Study of gut microbiota alterations in Alzheimer's dementia patients from Kazakhstan. Sci. Rep. 12:15115. doi: 10.1038/s41598-022-19393-0, 36068280 PMC9448737

[ref9] Krueger M. E. Boles J. S. Simon Z. D. Alvarez S. D. Mcfarland N. R. Okun M. S. . (2025). Comparative analysis of Parkinson’s and inflammatory bowel disease gut microbiomes reveals shared butyrate-producing bacteria depletion. NPJ Parkinsons Dis. 11:50. doi: 10.1038/s41531-025-00894-4, 40108151 PMC11923181

[ref10] Kulcsarova K. Bang C. Berg D. Schaeffer E. (2023). Pesticides and the microbiome-gut-brain Axis: convergent pathways in the pathogenesis of Parkinson's disease. J. Parkinsons Dis. 13, 1079–1106. doi: 10.3233/JPD-230206, 37927277 PMC10657696

[ref11] Li Y. Chen Y. Jiang L. Zhang J. Tong X. Chen D. . (2021). Intestinal inflammation and Parkinson's disease. Aging Dis. 12, 2052–2068. doi: 10.14336/AD.2021.0418, 34881085 PMC8612622

[ref12] Li W. Wu X. Hu X. Wang T. Liang S. Duan Y. . (2017). Structural changes of gut microbiota in Parkinson's disease and its correlation with clinical features. Sci. China Life Sci. 60, 1223–1233. doi: 10.1007/s11427-016-9001-4, 28536926

[ref13] Lindefeldt M. Eng A. Darban H. Bjerkner A. Zetterström C. K. Allander T. . (2019). The ketogenic diet influences taxonomic and functional composition of the gut microbiota in children with severe epilepsy. NPJ Biofilms Microb. 5:5. doi: 10.1038/s41522-018-0073-2, 30701077 PMC6344533

[ref14] Maini R. V. Bess E. N. Bisanz J. E. Turnbaugh P. J. Balskus E. P. (2019). Discovery and inhibition of an interspecies gut bacterial pathway for levodopa metabolism. Science 364:10.1126/science.aau6323:6323. doi: 10.1126/science.aau6323 31196984 PMC7745125

[ref15] Menozzi E. Schapira A. H. V. Borghammer P. (2025). The gut-brain axis in Parkinson disease: emerging concepts and therapeutic implications. Mov. Disord. Clin. Pract. 12, 904–916. doi: 10.1002/mdc3.70029, 40079755 PMC12275011

[ref16] Nuli R. Cai J. Kadeer A. Zhang Y. Mohemaiti P. (2019). Integrative analysis toward different glucose tolerance-related gut microbiota and diet. Front. Endocrinol. 10:295. doi: 10.3389/fendo.2019.00295, 31191448 PMC6546033

[ref17] Olson C. A. Vuong H. E. Yano J. M. Liang Q. Y. Nusbaum D. J. Hsiao E. Y. (2018). The gut microbiota mediates the anti-seizure effects of the ketogenic diet. Cell 173, 1728–1741.e13. doi: 10.1016/j.cell.2018.04.027, 29804833 PMC6003870

[ref18] Parada V. D. la De Fuente M. K. Landskron G. González M. J. Quera R. Dijkstra G. . (2019). Short chain fatty acids (SCFAs)-mediated gut epithelial and immune regulation and its relevance for inflammatory bowel diseases. Front. Immunol. 10:277. doi: 10.3389/fimmu.2019.00277 30915065 PMC6421268

[ref19] Qian Y. Yang X. Xu S. Huang P. Li B. Du J . (2020). Gut metagenomics-derived genes as potential biomarkers of Parkinson's disease. Brain 143, 2474–2489. doi: 10.1093/brain/awaa201, 32844199

[ref20] Reichardt N. Duncan S. H. Young P. Belenguer A. Mcwilliam L. C. Scott K. P. . (2014). Phylogenetic distribution of three pathways for propionate production within the human gut microbiota. ISME J. 8, 1323–1335. doi: 10.1038/ismej.2014.14 24553467 PMC4030238

[ref21] Scheperjans F. Aho V. Pereira P. A. Koskinen K. Paulin L. Pekkonen E. . (2015). Gut microbiota are related to Parkinson's disease and clinical phenotype. Mov. Disord. 30, 350–358. doi: 10.1002/mds.26069, 25476529

[ref22] Steinmetz J. D. Seeher K. M. Schiess N. Nichols E. Cao B. Servili C. . (2024). Global, regional, and national burden of disorders affecting the nervous system, 1990–2021: a systematic analysis for the global burden of disease study 2021. Lancet Neurol. 23, 344–381. doi: 10.1016/S1474-4422(24)00038-3, 38493795 PMC10949203

[ref23] Tidman M. M. White D. White T. (2022). Effects of an low carbohydrate/healthy fat/ketogenic diet on biomarkers of health and symptoms, anxiety and depression in Parkinson's disease: a pilot study. Neurodegener. Dis. Manag. 12, 57–66. doi: 10.2217/nmt-2021-0033, 35179078

[ref24] van der Louw E. van den Hurk D. Neal E. Leiendecker B. Fitzsimmon G. Dority L. . (2016). Ketogenic diet guidelines for infants with refractory epilepsy. Eur. J. Paediatr. Neurol. 20, 798–809. doi: 10.1016/j.ejpn.2016.07.009, 27470655

[ref25] van Kessel S. P. Frye A. K. El-Gendy A. O. Castejon M. Keshavarzian A. van Dijk G. . (2019). Gut bacterial tyrosine decarboxylases restrict levels of levodopa in the treatment of Parkinson's disease. Nat. Commun. 10:310. doi: 10.1038/s41467-019-08294-y, 30659181 PMC6338741

[ref26] Vanitallie T. B. Nonas C. Di Rocco A. Boyar K. Hyams K. Heymsfield S. B. (2005). Treatment of Parkinson disease with diet-induced hyperketonemia: a feasibility study. Neurology 64, 728–730. doi: 10.1212/01.WNL.0000152046.11390.45, 15728303

[ref27] Vidal-Martinez G. Chin B. Camarillo C. Herrera G. V. Yang B. Sarosiek I. . (2020). A pilot microbiota study in Parkinson's disease patients versus control subjects, and effects of FTY720 and FTY720-Mitoxy therapies in parkinsonian and multiple system atrophy mouse models. J. Parkinsons Dis. 10, 185–192. doi: 10.3233/JPD-191693, 31561385 PMC7029363

[ref28] Wang Y. Tong Q. Ma S. R. Zhao Z. X. Pan L. B. Cong L. . (2021). Oral berberine improves brain dopa/dopamine levels to ameliorate Parkinson's disease by regulating gut microbiota. Signal Transduct. Target. Ther. 6:77. doi: 10.1038/s41392-020-00456-5, 33623004 PMC7902645

[ref29] Xu H. M. Huang H. L. Liu Y. D. Zhu J. Q. Zhou Y. L. Chen H. T. . (2021). Selection strategy of dextran sulfate sodium-induced acute or chronic colitis mouse models based on gut microbial profile. BMC Microbiol. 21:279. doi: 10.1186/s12866-021-02342-8, 34654370 PMC8520286

[ref30] Zhang F. Yue L. Fang X. Wang G. Li C. Sun X. . (2020). Altered gut microbiota in Parkinson's disease patients/healthy spouses and its association with clinical features. Parkinsonism Relat. Disord. 81, 84–88. doi: 10.1016/j.parkreldis.2020.10.034, 33099131

